# Hidden community interlayer spillover detection in financial multilayer networks: Generalization of hierarchical clustering to multilayer networks

**DOI:** 10.1371/journal.pone.0330372

**Published:** 2025-09-03

**Authors:** Jamshid Ardalankia, Ali Habibnia, Marcel Ausloos, G. Reza Jafari

**Affiliations:** 1 Department of Economics, Virginia Tech, Blacksburg, Virginia, United States of America; 2 Dataism Laboratory for Quantitative Finance, Virginia Tech, Blacksburg, Virginia, United States of America; 3 School of Business, University of Leicester, Brookfield, Leicester, United Kingdom; 4 Department of Statistics and Econometrics, Bucharest University of Economic Studies, Bucharest, Romania; 5 Group of Researchers Applying Physics in Economy and Sociology (GRAPES), Beauvallon, Liège Angleur, Belgium; 6 Universitatea Babeş-Bolyai, Cluj-Napoca, Romania; 7 Department of Physics, Shahid Beheshti University, Evin, Tehran, Iran; 8 Center for Communications Technology, London Metropolitan University, London, United Kingdom; 9 Chandigarh Group of Colleges Jhanjeri, Punjab, India; Central Bank of Brazil, BRAZIL

## Abstract

Interdependent networks structurally influence each other so that the source network imposes hidden community structures into the target network. We propose a mathematical model so that when introducing an interlayer similarity function we generalize hierarchical clustering approaches for multilayer networks. The proposed methodology shows how a “source” network influences the “target” network via structural spillovers that are hidden and are not detectable by conventional community detection methods. The methodology reveals evidence that hidden interlayer interactions consequently generate hidden links on the target network. These hidden links construct hidden community structures on the target network (imposed from the source network) that are distinct from the community structures of the solo target network (without the presence of the source network). This model applies to systems with hidden interlayer interactions, such as, e.g., covert criminal groups, inter-platform social network interactions, scientific research groups, and financial markets. Financial markets are well known for complicated endogenous and exogenous, but often hidden, not to say the least, asymmetric layer interactions. We implement our model on multilayer financial networks: in particular, we find that trading value logarithmic changes (source) impose hidden community structures on the price return network (target). The main finding is that adding another relevant layer, such as the trading value layer, adds more information to systemic behaviors throughout the price return network. Dismissing it may yield less systemic information and underestimation of systemic risk because the footprint of some structures on the target network originated from another layer and is not detectable by singling out the target layer. As an empirical application, we exploit the methodology to define another perspective on portfolio diversification.

## Introduction

In a complex network, beyond the visible links, there may exist hidden connections that play a crucial role in shaping the network’s structure and its communities. These hidden links are not directly observable through simple node-to-node interactions [1–3]. Moreover, network-to-network interactions can generate some community structures that are hidden from the eyes or conventional community detection methods. The necessity of evaluating how two networks are connected, interacting, and influencing each other is highlighted by the limitations of studying isolated networks. Yet, real-world systems are interconnected and must be modeled as interdependent networks to understand their behavior accurately. Ignoring the interactions between networks can lead to incomplete or misleading conclusions about network robustness and connectivity [4], such as cascading failures [5] in interacting and interdependent networks [6–8]. One of the shortcomings of the single-layer analysis of node-to-node connections is dismissing the detection of structural effects that two networks impose (or induce) on (in) each other. Many network systems are intrinsically multilayer networks [9–11] with covert interlayer interactions, making these networks appearing as a single-layer. In such systems, members may actively cooperate within the formerly dismissed (source) layer and use the source layer to rule their behaviors in the observable (target) layer. They manage, form their communities, and guide their behaviors as a group in the target layer without leaving a trace for identification in the target layer. Thus, the footprints of *covert communities* in the target layer do not automatically exist in the target layer but are “suggested” because of the source layer. Hence, with covert interlayer interactions, the spillover of community structures from the source layer onto the target layer might occur. Hence, there is no guarantee that “all” community information of the target layer exists solely within the target layer itself. Accordingly, conventional community detection methods cannot capture such imposed (or induced) communities. To capture all community information within the target layer, one must investigate how community structures on the source layer impose (or induce) community structures onto the target layer. Conventional community detection methods work well based on the highlighted and significant interactions, but the covert groups leave no trace of their members’ relationships on the target layer.

Our contribution proposes a new mathematical model to indicate how two interacting networks influence each other via their community structures and generate interlayer structural spillovers. Finding the community structures on a target network that are generated due to the lack of independence from the interacting source and target networks is not feasible without the source network. We show how adding layers to the analysis provides more structural information within a network. The source network induces some of its structures in an interlayer manner to impose and induce an *a priori* invisible community structure on the target network. The new invisible community structures on the target network are not unique and are different from the community structures in the target network without the presence of the source network. This reveals the presence and the intensity of internetwork structural spillover that the source network transmits to the target network. Our model has significant implications for compliance purposes, anti-terrorism, anti-money laundering measures, but also (research) team ranking in academic fields, and systemic risk evaluation in financial-economic networks.

As an empirical application, we use hierarchical clustering to assess investment portfolio diversification by finding the interlayer effects from the trading value network onto the price network. We represent the hidden community structures in the price return network (target) that originate from the trading value network (source). We show that, for a given level of portfolio diversification on the price return layer, trading value–price interlayer interactions induce a range of effective diversification levels within the price return layer.

Another potential application is social network analysis. Relationships with friends on Facebook, X, WhatsApp, and text messaging may differ; one might share political beliefs with a group of friends on X, use Facebook for entertainment, and WhatsApp or text messages for daily communications. Consequently, the communities that an individual belongs to on Facebook, X (Twitter), and WhatsApp may differ in essence, but the quality of relationships on one platform can influence relationships on another. The same thing may hold in online-offline (face-to-face) interactions, such that spillover effects may transmit the dynamics through internetwork interactions (from online to face-to-face networks and vice versa) [12]. Thus, the individuals form their communities on an online layer and perform their activities on another layer. Well-known examples of such a phenomenon are security services or terrorist groups that raised a significant global concern and need to be resolved [13]. For instance, ISIS designed and managed its terrorist communities on video game platforms [15] (source layer). The realization of their leadership in online gaming (source layer) took place in the real world (target layer). The interaction among these two layers was hardly detectable because the single-layer analysis of node-to-node connections dismissed the detection of structural effects that the two networks impose on each other. The conventional community detection methods on the target layer may not necessarily find all structures within the target layer because the footprints of such structures are located on the source layer, not in the target layer itself. The community structures in the target layer, without considering communities on the source layer, are hidden at first but have the power to form and lead their groups in the target layer.

In the same line of thought, it is of common knowledge that (academic) research groups form communities because of the specificity of their research fields, but may belong to other communities because of the multidisciplinary essence of their research, not to mention external causes like geopolitical and economic constraints.

Furthermore, studies indicate that online gaming networks may serve as a haven for money laundering due to inadequate monitoring [14]. Criminals can register for these games at no cost, purchase substantial amounts of in-game currency using stolen funds, and subsequently sell it to other players or on digital platforms in exchange for cryptocurrency. Also, by using microtransactions, they avoid being detected since small amounts do not raise alarms like large money transfers [16]. Plus, criminals coordinate and communicate through “party chat [17]” (source layer) in multiplayer online role-playing to evade detection by authorities, yet it has consequences on the target layer. Criminal and terrorist groups such as ISIS, fraud, and money laundering groups know their members and have a high rate of interactions and collaboration on other layers or platforms [13–16], but they hide their interactions from the eyes of security services to be invisible within visible layers. The structural behavior of members (nodes) is how they participate in the network via links. Having hidden connections in some layers, the structural behavior of members is dismissed by the authorities, authorities may not be able to identify organized criminals. Since the source layer is responsible for some covert structures within the target layer, the similarity between the establishing behavior of a node throughout different layers conveys fundamental information about the whole structure. The conventional community detection methods are not built to find such transactions because they work well based on significant and highlighted connections.

Several research papers have recently conducted methods to differentiate between topological and structural financial phenomena. For instance, crisis analysis by topological features of multi-level resolution of communities [18], the effects of inter-community links on the phase transition from stability toward instability of a network [32]. By constructing a network via mapping the amplitudes into nodes and then mapping the difference between amplitudes into links, they clustered between two highly coupled and two weakly coupled time series by statistical and topological features of the constructed networks [33]. Other works include community structures in the Pearson correlation network of credit default swap for portfolio risk modeling [34], the short-term and long-term changes of community structures in big market movements [19], and identifying the persistence of community structure in a multilayer network of time-dependent financial asset correlation [26].

### Definitions

**Network definition.** We define a multiplex network ℳ, with 2 layers as a tuple of undirected, weighted graphs $\left(G,G^{\prime }\right)$, respectively representing the target and source layers. G=(V,E) is the target layer influenced by the structure of the source layer. $G^{\prime }=(V,E^{\prime })$ is the source layer that influences the structure of the target layer. The network has the following properties. $V=\{v_{1},v_{2},\ldots ,v_{N}\}$ is a common node set shared across both layers. $A,A^{\prime }\in {\mathbb{R}}^{N\times N}$ are the weighted symmetric adjacency matrices of *G* and $G^{\prime }$, respectively. Each node $v_{i}\in V$ is associated with two real-valued time series of length *T*. The timeseries corresponding to node *i* on the target and source are ${𝐱}_{i}=(x_{i,1},x_{i,2},\ldots ,x_{i,T})$ and ${𝐱}_{i}^{\prime }=(x_{i,1}^{\prime },x_{i,2}^{\prime },\ldots ,x_{i,T}^{\prime })$, respectively. Link weights are computed from the Pearson correlation coefficient (the network is given). Thus, the link weights for the target and the source are $w_{ij}=\rho ({𝐱}_{i},{𝐱}_{j})$ and $w_{i^{\prime }j^{\prime }}=\rho ({𝐱}_{i}^{\prime },{𝐱}_{j}^{\prime })$, respectively. Accordingly, the adjacency matrices for the target and source are defined by *A* and $A^{\prime }$ such that $A_{ij}=w_{ij}$ and $A_{i^{\prime }j^{\prime }}=w_{i^{\prime }j^{\prime }}$, respectively. Intralayer link sets are given by $E=\{(v_{i},v_{j})\in V\;\times \;V:A_{ij}\neq 0\}$ and $E^{\prime }=\{(v_{i^{\prime }},v_{j^{\prime }})\in V\;\times \;V:A_{i^{\prime }j^{\prime }}\neq 0\}$. There are no explicit observable interlayer links, $E^{\left[\text{interlayer}\right]}=\emptyset$.

**Fig 1:**
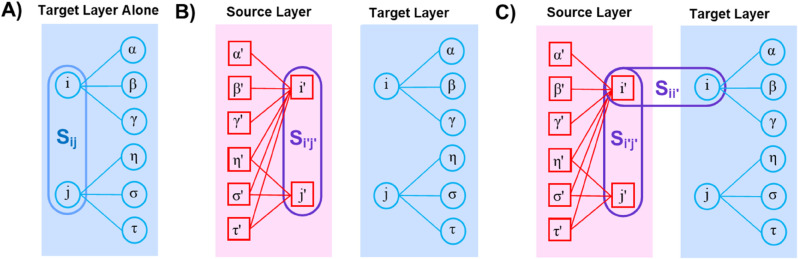
Definitions. This figure schematically shows the configuration of single-layer **(Panel A)**, multilayer **(Panel B)**, and their involvement via structural similarity **(Panel C)**. The target and source layers contain nodes *i*, *j*, *α*, *β*, *γ*, *σ*, and *τ* ($i^{\prime }$, $j^{\prime }$, $\alpha^{\prime }$, $\beta^{\prime }$, $\gamma^{\prime }$, $\sigma^{\prime }$, and $\tau^{\prime }$). The links are shown by solid straight lines connecting the nodes. **Panel A** schematically demonstrates the target layer in a single-layer setting containing nodes *i*, *j*, *α*, *β*, *γ*, *σ*. There exists conventional intralayer structural similarity *S*_*ij*_ between each pair of nodes *i* and *j* on the isolated layer. **Panel B** demonstrates that there can be another layer in the system–as the source layer– and it also possesses intralayer structural similarity $S_{i^{\prime }j^{\prime }}$ in its own right in multilayer settings. **Panel C** shows the components of interlayer structural similarity, $S_{ij^{\prime }}$, between nodes *i* and *j*. These structural similarities identify the community membership of each node.

**Structural similarity measures.** Let $𝒮=[S_{ij}]$ (Fig 1A) and ${𝒮}^{\prime }=[S_{i^{\prime }j^{\prime }}]$ (Fig 1B) denote the pairwise intralayer structural similarity matrices derived from the target and source layers. Let ${𝒮}^{\left[\text{inter}\right]}=[S_{ij^{\prime }}]$ represent the interlayer structural similarity matrix obtaining node topologies across layers. Since isolated nodes do not establish any links, they are not participating in the network. Thus, their similarity with all other nodes is zero, as there are no links to determine whether they are similar or not. The vector of node-wise interlayer self-similarities is denoted by ${𝐬}^{\left[\text{inter}\right]}:=[S_{ii^{\prime }}]=(S_{11^{\prime }},S_{22^{\prime }},\ldots ,S_{NN^{\prime }})$. A hierarchical clustering operator ℋ(·,·) gets the structural similarity matrix and the layer to provide an updated structural organization (clustering heatmap) on the layer such that ${CH}^{\prime }:=ℋ({𝒮}^{\prime },G^{\prime }),CH:=ℋ(𝒮,G),{CH}^{\left[\text{inter}\right]}:=ℋ({𝒮}^{\left[\text{inter}\right]},G)$. Here, ${CH}^{\prime }$ and CH capture the mesoscopic structure within each layer, while ${CH}^{\left[\text{inter}\right]}$ encodes the mesoscopic structure between corresponding nodes across layers. In this paper, we propose a mathematical model for ${CH}^{\left[\text{inter}\right]}$ by using ${𝒮}^{\left[\text{inter}\right]}$ aiming to discover interlayer structural spillover from the source layer into the target layer.

**Clustering heatmap.** The clustering heatmap is obtained from hierarchical clustering based on calculating the structural similarity function of a correlation matrix. Hierarchical clustering uses the similarity matrix to arrange the nodes on the correlation heatmap so that nodes with high similarity are placed close to each other. In this paper, we investigate three clustering heatmaps, ${CH}^{\prime }:=ℋ({𝒮}^{\prime },G^{\prime })$ on the source layer $G^{\prime }$ using the intralayer structural similarity of the source (${𝒮}^{\prime }$), CH:=ℋ(𝒮,G) on the target layer *G* using the intralayer structural similarity of the target (𝒮), and ${CH}^{\left[\text{inter}\right]}:=ℋ({𝒮}^{\left[\text{inter}\right]},G)$ on the source layer *G* using the interlayer structural similarity (${𝒮}^{\left[\text{inter}\right]}$).

**Mapping.** This implies that a clustering heatmap of a layer organizes nodes based on a structural similarity matrix using hierarchical clustering. This can be done using the structural similarity and the correlation heatmap of the same layer, or it can be done using the source-induced structural similarity on the correlation heatmap of the target layer. In a multiplex setting, nodes on both layers are the same, but the intralayer relationships between the nodes on one layer may differ from the intralayer relationships of the nodes on another layer. These relationships and topological features construct community structures within layers. Since on each layer, there exists some structural information, mapping is when we use an obtained source-induced structural similarity to organize the nodes on the target layer, e.g. ${CH}^{\left[\text{inter}\right]}:=ℋ({𝒮}^{\left[\text{inter}\right]},G)$. Mapping is a node-to-node correspondence of the structural similarity matrix and *G*.

**Position or placement of nodes on the heatmap.** Clustering heatmap arranges nodes based on their corresponding similarity as the similar nodes are placed close compared with nodes that are not similar. Since the positions of the nodes across the axes of the clustering heatmaps contain the underlying information of the structural pattern of a layer, mapping the sequence (permutation) of nodes based on the similarity matrix induced by the source layer into the target layer unveils latent patterns for which the source network is responsible [53] on the target layer. This process provides an alternative classification setting that is based on invisible links that influence the structure of the systems [54].

**Covert hidden structures.** Covert hidden structures are the mesoscopic structure on the target layer that is obtained from $ℋ({𝒮}^{\left[\text{inter}\right]},G)$ and $ℋ({𝒮}^{\prime },G)$. Hidden communities are mapped from the source layer onto the target layer. Since the positions of the nodes across the axes of the heatmaps contain the underlying information of the structural pattern of the source network, mapping it to the target layer unveils latent patterns for which the source network is responsible [53]. This process provides an alternative classification setting that is based on invisible links that influence the structure of the systems [54]. The resulting hidden structure is not detectable by conventional clustering heatmap CH:=ℋ(𝒮,G) because conventional methods detect community structures using highlighted links. Also, since the hidden structure is induced by another layer, it is not detectable by the sole analysis of the target layer.

## Community detection with hierarchical clustering

Communities are made of nodes with high similarity and usually have a high density of links between them and a lower density of links between communities. Highly similar nodes are located in more similar clusters. Thus, in the graphical representation of node placements, nodes with higher similarity are closer to each other and are grouped together in the aftermath of clustering. Much work has been done on detecting communities in multilayer [26–29,36] and in single-layer networks [37–43] using similarity criteria [37,44–47]. The underlying concept consists of grouping nodes based on the maximum similarity of highlighted features and they mainly focus on detecting significant nodes and links and are based on the high number and weight of links. For instance, since covert organizations try to keep themselves hidden, they form structures by reducing the number and the weight of their links whence avoiding significant connections in the observable network. Normally, covert organizations use a less tangible environment (source network) to connect their individuals on the visible environment (target network). The result is some minimal communication on the observable network. In this way, they are hidden when using classically searching community monitoring methods.

Among community detection methods, hierarchical clustering [48,49] is one of the methods to identify the existing structures within a network. This method is already used on financial return correlations as an appropriate method for community detection in financial markets [8,44,50]. Hierarchical Clustering is an algorithm that creates a hierarchy of clusters from the similar nodes of a given network, merging nearest clusters until only one remains [40–42,44]. There are several alternatives for describing the pairwise associations of time series and nodes [33,37,44,50] by defining the similarity and distance measurements. These alternatives access the topological structure of the network with *N* nodes through an adjacent matrix *A*, and the degrees of the nodes, $K_{i}=\sum_{j=1}^{N}A_{ij}$ (1≤i≤N). Following [42], the (cosine) similarity *S*_*ij*_ between nodes *i* and *j* is:

$$S_{ij}=\frac{n_{ij}}{\sqrt{\sum_{k=1}^{N}A_{ik}^{2}}\sqrt{\sum_{k=1}^{N}A_{jk}^{2}}},\;0\leq S\leq 1$$ (1)

Here, $n_{ij}=\sum_{k=1}^{N}A_{ik}A_{jk}$ denotes the number of shared neighbors between nodes *i* and *j*, with the normalization factor in the denominator being the product of the degrees *K*_*i*_ and *K*_*j*_. In the context of weighted networks, $K_{i}=\Sigma_{j=1}^{N}A_{ij}$ represents the sum of weights associated with the links converging upon node *i*.

In comparison with other community detection methods on networks, hierarchical clustering has several advantages. It provides multi-level resolutions and detects multiscale communities [60], without needing to have an ad hoc number of communities [62]. Moreover, since hierarchical clustering is robust to small-scale noise, an approach addresses the resolution limit problem that modularity-based methods struggle with [63]. Hierarchical clustering is also fruitfully applicable in weighted networks [38,39].

## Mapping communities from the source layer into the target layer: Usefulness of added layers

Layers by nature are networks and the whole system is a nexus of interconnected networks [6–8]. Accordingly, the whole structure is typically composed of smaller interdependent networks (or layers), meaning that these may interact with each other. We consider a multiplex network with interacting layers and mutual nodes. Thus, the hypothesis is that a source layer influences the target layer by imposing community structures. Through this interlayer information, several hidden community structures in the target layer may become highlighted; throughout our research, we propose a mathematical model to find and explore these hidden community structures. Such hidden interlayer structural influences are not detectable without the presence of the source layer. We are all curious to find the origin of the spillover of community structures among layers. Thus, we must focus on how community structures on a target layer are imposed (or induced) from a source layer. In this paper, we discuss this question and show that in networks with interconnected layers, the community structure on the target network differs from the community structures found in the target network but in the absence of the source network. This reveals the presence and the intensity of interlayer information that the source layer transmits to the target layer. Rather than assuming that influences within a system can be reduced to a single type of link, notice that some models introduce a multilayer network [9–11] in which nodes are connected with various types of links [51]. However, in networks with interdependent layers, models based on single-layer pairwise interactions often fail to fully capture the intricate relationships between network nodes.

**Fig 2:**
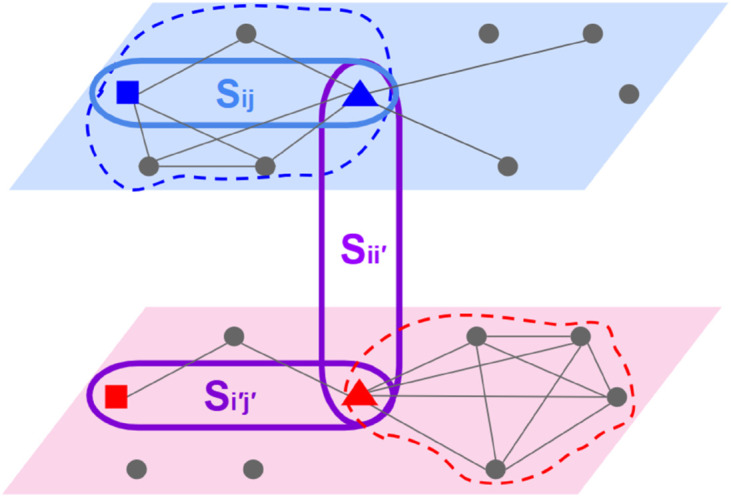
Schematic representation of intra- and interlayer node Similarities and Community Assignments in a Two-Layer Network. The target layer (top layer) and the source layer (bottom layer), together with nodes *i* and *j* (in blue triangle and square, respectively) on the target layer, and their corresponding representations, $i^{\prime }$ and $j^{\prime }$ (in red triangle and square, respectively) on the source layer are shown. The remaining nodes are shown in black solid circles. The links are shown in black solid lines. The communities on the target and source layers are shown in blue and red dashed closed curves. The figure demonstrates how node placement in communities based on different structural similarities is different across layers. *S*_*ij*_ (Eq 5) shows the intralayer similarity between nodes *i* and *j* on the target layer. Also, $S_{i^{\prime }j^{\prime }}$ (Eq 5) shows the intralayer similarity between nodes $i^{\prime }$ and $j^{\prime }$ on the source layer. $S_{ii^{\prime }}$ (Eq 4) shows the interlayer self-similarity between the two representations of node *i* on the two layers.

Fig 2 schematically shows the role of adding layers in revealing more structural information. This figure schematically contains two interacting layers with mutual nodes but different link types. Fig 2 shows that node *i* (solid triangles) can be placed into different communities. Its inclusion in either of the communities originates from the similarity matrix used to arrange the nodes in communities. Although any two nodes within a singular layer may exhibit a degree of similarity, these similarities can increase or decline within the architecture of multilayer networks.

In this research, we propose a new similarity function that finds some more hidden structures that originate from the source and spread into the target layer. Then, we use the proposed similarity function to map it onto the target layer and generate a new arrangement of community structures on the target layer. In this way, the positions of the nodes in the target layer correlation heatmap are based on the proposed similarities.

## Generalization of hierarchical clustering for multilayer networks

In systems with a multilayer nature, the structures in the source layer can impose some structures into the target layer. The conventional similarity measurements must be generalized to multilayer to encompass multilayer information within community detection methods. We extend hierarchical clustering into the domain of multilayer networks, thereby unmasking the covert structures that operate across layers bidirectionally.

In multiplex networks, nodes on the source and target layers are projections of each other, representing the same individuals. However, their connections within the source and target layers convey different information.

In a network with *N* nodes and the adjacency matrix *A*, the links of node *i* is represented by vector $|i\rangle =(A_{i1},A_{i2},\ldots ,A_{iN}){\;\;}^{T}$ and *A*_*ii*_ = 0 implies that there is no self-loop. For unweighted (binary) networks [42], we have $A_{ij}\in \{0,1\}$. In this case, $K_{i}=\sum_{j=1}^{N}A_{ij}$ is the degree of node *i* and $n_{ij}=\sum_{k}A_{ik}A_{jk}$ denotes the number of shared neighbors between nodes *i* and *j*.

The pattern of connections around two nodes provides key insights into how structurally similar the two nodes truly are in terms of establishing weighted links (as a result of correlations) with others. Thus, understanding node similarity requires looking at both the presence of connections and the weight of those connections.

We first add weight attributes to links to include the weights of connections, and we have $A_{ij}\in [0,1]$. Thereby, $n_{ij}=\sum_{k}A_{ik}A_{jk}$ measures how strongly the nodes *i* and *j* are positively or negatively associated with other nodes such as *k*. In our model, it is not just about whether a pair of nodes *i* and *j* are connected with other nodes, but also how strong they are positively or negatively associated with others. When the topology of attachments from node *i* and *j* to other nodes are the same, the nodes *i* and *j* are structurally similar. One can represent the connections of node *i* with the vector $\left\langle i|=(A_{i1},A_{i2},\ldots ,A_{iN}\right)$. In the cosine similarity method [37,42], by normalizing the vector corresponding to connections of node *i*, we have:

$$|i\rangle_{normalized}=\frac{1}{\sqrt{\sum_{k=1}^{N}A_{ik}^{2}}}\left(\begin{array}{c} A_{i1}\\ A_{i2}\\ \vdots \\ A_{iN} \end{array}\right).$$ (2)

According to Fig 2, the intralayer (within-layer) structural similarity of the nodes *i* and *j* in a weighted network is:

$$S_{ij}=\langle i|j\rangle =\frac{\sum_{k}A_{ik}A_{jk}}{\sqrt{\sum_{k}A_{ik}^{2}}\sqrt{\sum_{k}A_{jk}^{2}}}=\frac{n_{ij}}{\sqrt{\sum_{k}A_{ik}^{2}}\sqrt{\sum_{k}A_{jk}^{2}}}.$$ (3)

As a general rule in structural similarity [37–39], *n*_*ij*_ for binary links counts the number of shared neighbors between node *i* and *j*. Then, to assess the ratio of the mutual connections from all connections that nodes *i* and *j* establish in the structure, *n*_*ij*_ is scaled by the denominator, which is the norm of all links of *i* multiplied by the norm of all links of *i*.

We introduce Eq 4, interlayer self-similarity of node *i*, to measure how much the behavior of node *i* in establishing links is similar on the source and target layers.

By Eq 4, one can detect the interlayer structural similarity between those layers via node *i*. A higher similarity in making weighted links yields more information flow between those nodes. Thereby, the first step is to observe the interlayer similarity between the behaviors of node *i* in making connections throughout different layers:

$$S_{ii^{\prime }}=\frac{\sum_{k,k^{\prime }}A_{ik}A_{i^{\prime }k^{\prime }}}{\sqrt{\sum_{k}A_{ik}^{2}}\sqrt{\sum_{k^{\prime }}A_{i^{\prime }k^{\prime }}^{2}}}=\frac{n_{ii^{\prime }}}{\sqrt{\sum_{k}A_{ik}^{2}}\sqrt{\sum_{k^{\prime }}A_{i^{\prime }k^{\prime }}^{2}}},$$ (4)

where $n_{ii^{\prime }}$ is the dot product of row *i* of the adjacency matrix *A* (the target layer) and row $i^{\prime }$ of adjacency matrix $A^{\prime }$ (the source layer). $n_{ii^{\prime }}$ measures the structural similarity between the two reflections of node *i* on both layers in establishing connections. If $n_{ii^{\prime }}$ is high, it implies that when node *i* on the target layer is attached to the nodes *α*, *β*, and *γ*, then its representation on the source is attached to the nodes $\alpha^{\prime }$, $\beta^{\prime }$, and $\gamma^{\prime }$ with the same weights as the target: $A_{i,\alpha }=A_{i^{\prime },\alpha^{\prime }},A_{i,\beta }=A_{i^{\prime },\beta^{\prime }},A_{i,\gamma }=A_{i^{\prime },\gamma^{\prime }}$ (see the Appendix). $n_{ii^{\prime }}$ represents the configuration of connecting to shared neighbors between node *i* in the source layer and node $i^{\prime }$ in the target layer. This value captures how structurally similar a node’s connectivity pattern is across layers.

Using structural similarity helps to find the community structures. If node *i* and *j* have a high similarity, it implies that nodes *i* and *j* make connections with the same nodes with similar link values. The intralayer similarity of nodes *i* and the intralayer similarity of nodes *j* in the source and target layer are as below, respectively:

$$S_{ij}=\frac{n_{ij}}{\sqrt{\sum_{k}A_{ik}^{2}}\sqrt{\sum_{k}A_{jk}^{2}}},\;S_{i^{\prime }j^{\prime }}=\frac{n_{i^{\prime }j^{\prime }}}{\sqrt{\sum_{k^{\prime }}A_{i^{\prime }k^{\prime }}^{2}}\sqrt{\sum_{k^{\prime }}A_{j^{\prime }k^{\prime }}^{2}}},$$ (5)

where *S*_*ij*_ and $S_{i^{\prime }j^{\prime }}$ measure how much the nodes *i* and *j* (the nodes $i^{\prime }$ and $j^{\prime }$) possess the same quality in establishing connections with other nodes within the source (target) layer. In other words, *S*_*ij*_ and $S_{i^{\prime }j^{\prime }}$ measure how nodes *i* and *j* establish similar links with similar nodes within their corresponding layer.

The next step is connecting the interlayer self-similarity of node *i* and intralayer similarity between node $i^{\prime }$ and $j^{\prime }$ on the source layer. By definition, as a general rule, the maximum similarity value is 1. We have:

$$S_{ij^{\prime }}=S_{ii^{\prime }}\cdot S_{i^{\prime }j^{\prime }}\leq S_{i^{\prime }j^{\prime }}\;(S_{ii^{\prime }}\leq 1),$$ (6)

According to Eq 6, some scenarios need to be discussed:

- If the behavior of node *i* is the same in source and target layers ($S_{ii^{\prime }}=1$), the information flow between the source layer into the target layer through node *i* is high. Thus, the node *i* plays an important role in transmitting community information from the source layer into the target layer ($S_{ij^{\prime }}=1\;\times \;S_{i^{\prime }j^{\prime }}$). Thus, if *S*_*ij*_ is high, the source-induced interlayer similarity into the target layer becomes high.
- If $S_{ii^{\prime }}=0$, the way that node *i* connects to other nodes on the source layer is by far different from that on the target layer. The way that node *i* is connected to other nodes on both layers does not follow similar patterns. Thus, the two reflections of node *i* on the two layers are independent in terms of link formation. This circumstance leads to $S_{ij^{\prime }}=0$.
- $S_{ij^{\prime }}$ and $S_{i^{\prime }j}$ take into account the interlayer structural similarity of *i* and *j*. Thus, since $S_{ii^{\prime }}$ and $S_{jj^{\prime }}$ are not necessarily equal, $S_{ij^{\prime }}$ and $S_{i^{\prime }j}$ are not the same.

**Fig 3:**
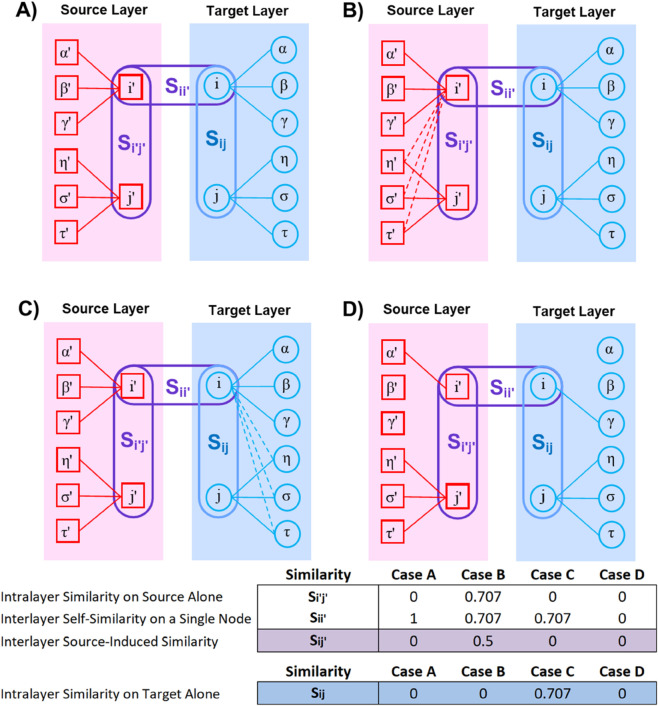
Toy Model Example. Four case studies that demonstrate how source structures may or may not yield hidden similarity structures between *i* and *j*: interlayer (between-layer) vs. intralayer (within-layer). For simplicity, we consider binary links. There are 8 nodes on the target and their representation on the source. The links are shown with solid and dashed lines.

For further investigation of the methodology, please refer to the toy model example, Fig 3, and Algorithm 1. To generalize the similarity between layers (Eq 6) for networks with more than two layers, the similarity between all the corresponding nodes in source layers and the target layer is formalized. For all nodes i∈Target Layer, and for all nodes $i^{\prime },j^{\prime }\in \text{ Source Layer(s)},\;\mathrm{and}\;i^{\prime },j^{\prime }\notin \text{Target Layer}$, $S_{ij^{\prime }}$ results in structural similarity between nodes *i* and *j* generated from the source layers. Intuitively, the method compares how similar node *i* is to other nodes (such as *j*) under the influence of multiple source layers. We have:

$$S_{ij^{\prime }}=\sum_{\begin{array}{c} i\in \text{Target Layer}\\ i^{\prime },j^{\prime }\in \text{Source Layer(s)} \end{array}}S_{ii^{\prime }}\cdot S_{i^{\prime }j^{\prime }}$$ (7)

**Algorithm 1 Interlayer community structure spillover.**

A multiplex network ℳ, with 2 layers as a tuple of undirected, weighted graphs $\left(𝐆,G^{\prime }\right)$.

𝐆=(𝐕,𝐄)

$G^{\prime }=(𝐕,E^{\prime })$

**Input:**

$𝐕=\{v_{1},v_{2},\ldots ,v_{N}\}$ is a common node set shared across both layers.

On the source layer: i,j,k,…; and their reflections on the target layer: $i^{\prime },j^{\prime },k^{\prime },\ldots$

Each node $v_{i}\in 𝐕$ is associated with two real-valued time series of length *T*.

${𝐱}_{i}=(x_{i,1},x_{i,2},\ldots ,x_{i,T})$     ⊳ Timeseries for the target layer

${𝐱}_{i}^{\prime }=(x_{i,1}^{\prime },x_{i,2}^{\prime },\ldots ,x_{i,T}^{\prime })$     ⊳ Timeseries for the source layer

**Output:**

${CH}^{\left[\text{inter}\right]}:=ℋ({𝒮}^{\left[\text{inter}\right]},G)$

1: **Input:** ${𝐱}_{i}$, ${𝐱}_{i}^{\prime }$ for all $v_{i}$ in $𝐕=\{v_{1},v_{2},\ldots ,v_{N}\}$

2: $A,A^{\prime }\in {\mathbb{R}}^{N\times N}$

3: Initialize 𝐀=zeros(N,N)

4: Initialize $A^{\prime }=zeros(N,N)$

5: **for** All nodes in **V do**

6:   $w_{ij}=\rho ({𝐱}_{i},{𝐱}_{j})$

7:   $w_{i^{\prime }j^{\prime }}=\rho ({𝐱}_{i}^{\prime },{𝐱}_{j}^{\prime })$

8:   $𝐀=[A_{ij}=w_{ij}]$

9:   $A^{\prime }=[A_{i^{\prime }j^{\prime }}=w_{i^{\prime }j^{\prime }}]$

10: **end for**

11: 𝐆= CreateLayer(Nodes= **V**, Links= **A**)

12: $G^{\prime }=$ CreateLayer(Nodes= **V**, Links=$A^{\prime }$)

13: **for** each node $\left(k,k^{\prime }\right)$ in **V** of $\left(𝐆,G^{\prime }\right)$ **do**

14:   **for** each node $\left(i,i^{\prime }\right)$, i≠k, $i^{\prime }\neq k^{\prime }$ in **V** of $\left(𝐆,G^{\prime }\right)$ **do**

15:    $n_{ii^{\prime }}=\sum A_{ik}A_{i^{\prime }k^{\prime }}$

16:    $S_{ii^{\prime }}=\frac{n_{ii^{\prime }}}{\sqrt{\sum_{k}A_{ik}^{2}}\sqrt{\sum_{k}A_{i^{\prime }k^{\prime }}^{2}}}$    ⊳ Eq 4: Interlayer Similarity

17:   **end for**

18:   **for** each node $\left(j,j^{\prime }\right)$, j≠k, $j^{\prime }\neq k^{\prime }$, j≠i, $j^{\prime }\neq i^{\prime }$ in **V** of

$\left(𝐆,G^{\prime }\right)$ **do**

19:    $n_{ij}=\sum A_{ik}A_{jk}$

20:    $S_{ij}=\frac{n_{ij}}{\sqrt{\sum_{k}A_{ik}^{2}}\sqrt{\sum_{k}A_{jk}^{2}}}$     ⊳ Eq 3: Intralayer Similarity on

Target

21:    $n_{i^{\prime }j^{\prime }}=\sum A_{i^{\prime }k^{\prime }}A_{j^{\prime }k^{\prime }}$

22:    $S_{i^{\prime }j^{\prime }}=\frac{n_{i^{\prime }j^{\prime }}}{\sqrt{\sum_{k^{\prime }}A_{i^{\prime }k^{\prime }}^{2}}\sqrt{\sum_{k^{\prime }}A_{j^{\prime }k^{\prime }}^{2}}}$    ⊳ Eq 5: Intralayer Similarity

on Source

23:    $S_{ij^{\prime }}=S_{ii^{\prime }}\times S_{i^{\prime }j^{\prime }}$      ⊳ Eq 6: Interlayer Similarity of *i* and

*j*

24:   **end for**

25:   ${𝒮}^{\left[\text{inter}\right]}=[S_{ij^{\prime }}]$

26: **end for**

27: ${CH}^{\left[\text{inter}\right]}=ℋ({𝒮}^{\left[\text{inter}\right]},𝐆)$

The influence of the source layer on the target layer arises from the changes in node similarity. This change in similarity leads to a reformation of the communities in the target layer compared to when the target layer relies solely on its own internal similarities. The root of the spillover from one layer to another lies in the fact that node similarity, which was previously computed solely within the target layer, now also arises from the source layers. As a result, we observe more community structures: one based on the internal similarity of the layer itself, and another formed by both the internal and interlayer similarities.

### Toy model

In this section, we investigate a hypothetical model showing the main different cases that may happen between *i* and *j*. Two types of local configurations make *n*_*ij*_ small. First, small *n*_*ij*_ happens while *i* and *j* have no common links, which in turn leads to *n*_*ij*_ = 0. Alternatively, *i* and *j* connect to the same assets with links with opposite signs.

Here, we discuss different scenarios that may happen in Fig 3. For simplicity, we use binary links to explain the calculation of case B as an example in the Appendix.

**Case A.** In panel A, structural similarity between $i^{\prime }$ and $j^{\prime }$ is zero because there are no mutual neighbors between them. Although the pattern of link formation of node *i* on both layers corresponds to each other, $S_{ij^{\prime }}=0$. Overall, whether one just takes into account the target layer (*S*_*ij*_) or both layers ($S_{ij^{\prime }}$), no systemic information is transmitted between nodes *i* and *j*.

**Case B.** In panel B, because of 3 mutual connections ($\eta^{\prime },\sigma^{\prime },\tau^{\prime }$) between $i^{\prime }$ and $j^{\prime }$, the structural similarity between node *i* and *j* on source layer is higher ($S_{i^{\prime }j^{\prime }}=0.7$). Also, comparing connections of node *i* on both layers, there exist 3 uncommon neighbors on both layers ($\eta^{\prime },\sigma^{\prime },\tau^{\prime }$), and accordingly, the structural similarity of node *i* between the two layers decreases ($S_{ii^{\prime }}=0.7$). Thus, $S_{ij^{\prime }}=0.5$. This is while observations solely based on the source layer do not show that nodes *i* and *j* have structural similarity, and accordingly, the conventional method is not able to show systemic transmission that occurs between *i* and *j*. Therefore, adding the source layer into the analysis adds more information on the systemic paths that may bridge *i* and *j*. Such a path is not detectable by the target alone and it is induced by the source.

**Case C.** In panel C, although the structural similarity between $i^{\prime }$ and $j^{\prime }$ on the source is zero, on the target, however, they have 3 mutual neighbors. This implies that $S_{ij^{\prime }}=0$ and *S*_*ij*_ = 0.7. Accordingly, for finding the structural similarity between *i* and *j* in this configuration, the source layer does not add any information, and the target solely is adequate.

**Case D.** In panel D, the way node *i* on the source layer behaves has nothing in common with the way it behaves on the target layer. Thereby, $S_{ii^{\prime }}=0$ and then $S_{ij^{\prime }}=0$. Hence, through node *i*, no interlayer structural spillover is transmitted from the source layer onto the target of asset *j*. In other words, node *i* does not play the role of an interlayer spillover.

## Financial data onto multilayer network

In this research, we use financial assets to illustrate our methodology. Multiple variables, such as price, trading volume, and trading value time series characterize financial assets. These can be modeled as multivariate time series, incorporating diverse variables (layers). There is a body of literature studying financial economic systems by monolayer and multilayer network perspective [6,20–25,33,35,39,40]. In this sense, there can be multiple types of connections (heterogeneous types of connections) between assets. The relationship between assets within one variable (source layer) may influence the relationship between assets within another variable (target layer). Since there are some coupled associations among different variables of multivariate financial time series [33,55], by putting the relationships of each variable within a layer and constructing its network, we aim to scrutinize the structural influence between these networks, and then, more structural information can be obtained by considering the structures imposed from a source layer.

Among community detection methods, hierarchical clustering [48,49] methods have been already used on financial return correlations [56–58] as an appropriate method for community detection in financial markets [8,44,50].

In our setting, each pair of assets has two types of connection: correlation between their price returns and correlation between logarithmic changes of their trading values. Investors use their money to trade stocks and enter the market through the trading value layer. The changes in trading value generate dynamics. In our research, the source layer is the trading value since the investors enter the market by exchanging cash for the stocks via this layer for a return on investment.

The price return layer is the target network since the final goal of investors is to gain profit from the price dynamics. The price return is the criterion for gain and loss in the market. Trading value at time *t* is the multiplication of the price of an asset at time *t* and the number of shares (trading volume) of that asset that are traded in that price at time *t*. Trading value indicates how much money is needed for a transaction of a certain number of shares at a certain price at time *t*. In this sense, trading value log. changes show the changes in the amount of cash flow used to trade the asset at a specific time. By that, we scrutinize how the trading value network induces its structures onto the price network. These new structures are the footprints of interlayer transmission of interdependencies between layers.

Our empirical investigation centers on the 204 firms publicly traded in the US from Nov. 2019 until Jan. 2024. We gathered data from a variety of US economic sectors based on the Global Industry Classification Standard (GICS). The GICS codes include 202020 (Industrials: Professional Services), 253020 (Consumer Discretionary: Diversified Consumer Services), 351020 (Health Care: Health Care Providers & Services), 401010 (Financials: Banks), 401020 (Financials: Thrifts & Mortgage Finance), 402020 (Financials: Consumer Finance), 402030 (Financials: Capital Markets), 402040 (Financials: Mortgage Real Estate Investment Trusts-REITs), 403010 (Financials: Insurance), 451020 (Information Technology: IT Services), 451030 (Information Technology: Software), 601010 (Real Estate: Equity Real Estate Investment Trusts-REITs), 601020 (Real Estate: Real Estate Management & Development). The combination of the firms in these industries is important because they are listed in the KBW index, S&P500-financials, NASDAQ-financials, and the KFT index (fintech firms). All time series are downloaded from Yahoo Finance.

In this work, two variables of price *p*(*t*) and trading value *tv*(*t*) are assigned to each asset. The price return time series is $x(t)=ln\frac{p(t+1)}{p(t)};t=1,2,\ldots ,T$. Time series for trading value logarithmic changes is tv(t)=p(t)×v(t);t=1,2,…,T, and $x^{\prime }(t)=ln\frac{tv(t+1)}{tv(t)}$, where *v*(*t*) and *tv*(*t*) stand for the time series of trading volume, and trading value, respectively. Since price return and logarithmic changes of trading value series are not the same variables, we subtract their average and divide them by their standard deviation to turn them into dimensionless series with one variance.

$$X(t)=\frac{x(t)-\bar{x}}{\sigma_{x}},\;X^{\prime }(t)=\frac{x^{\prime }(t)-\bar{x^{\prime }}}{\sigma_{x^{\prime }}}.$$ (8)

Now, we can define the layers in the multilayer network such that each layer conveys the correlation network underlying each variable. We construct the adjacency matrix of each layer by the correlation matrix. We make the diagonals zero to remove the effects of self-associations (self-loops) in the network such that $C(X_{i},X_{i})=C(X_{i}^{\prime },X_{i}^{\prime })=0$.

$$A_{ij}=C(X_{i},X_{j})=\frac{1}{N}\sum_{t=1}^{N}X_{i}(t)X_{j}(t),$$ (9)

$$A_{ij}^{\prime }=C(X_{i}^{\prime },X_{j}^{\prime })=\frac{1}{N}\sum_{t=1}^{N}X_{i}^{\prime }(t)X_{j}^{\prime }(t).$$ (10)

The correlation matrices in Eq 10 are used for the community detection steps and obtain hierarchical clustering heatmaps.

**Fig 4:**
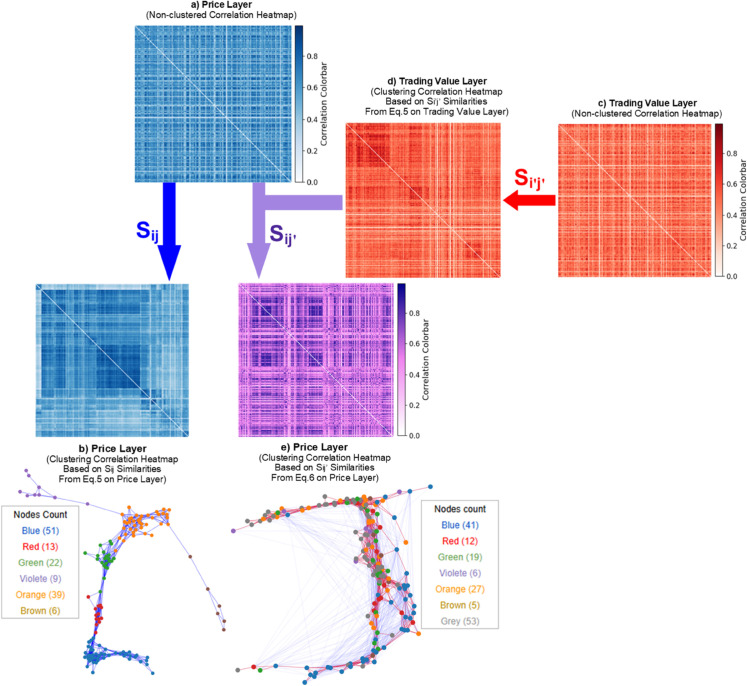
*S*_*ij*_ vs. $S_{ij^{\prime }}$. **Panel (a) and (c):** The blue and red heatmaps, respectively, represent the correlation heatmap of the price layer (target) and the correlation heatmap of the trading value layer (source) before community detection on either of them. **Panel (b):** The clustering heatmap with hierarchical clustering solely based on the price return within-layer similarities (*S*_*ij*_) as the target layer is implicated. **Panel (d):** The hierarchical clustering heatmap solely based on the trading value within-layer similarities ($S_{i^{\prime }j^{\prime }}$) as the source layer is implicated. **Panel (e):** After obtaining $S_{ij^{\prime }}$ and reordering the position of assets on the price layer heatmap, a piece of new structural information emerges in the price layer–which is different from the community structures on the price layer without the presence of trading value layer in the analysis, in panel (b). It shows that communities within the trading value layer can impose some structural information into the price layer.

## Results and discussion

**Fig 5:**
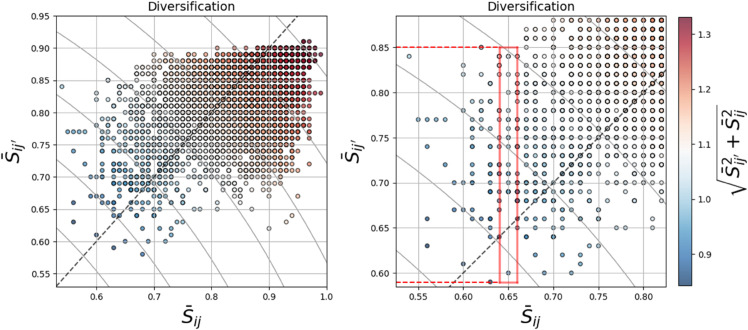
Intralayer Similarity vs. Interlayer Similarity. On the scatter plots, each dot represents a synthetic portfolio constructed from the real dataset. The X-axes show the within-layer price structural similarities of the portfolios (for the conventional diversification method), and the Y-axes demonstrate the between-layer similarities, which are the trading value-induced structural similarities onto the price layer (our proposed method). **Panel a)** If a portfolio is located on the angle bisector (dashed gray line), both methods result in the same result (${\bar{S}}_{ij^{\prime }}={\bar{S}}_{ij}$). **Panel b)** The zoomed-inset of the scatter plot is shown for a specific value of ${\bar{S}}_{ij}$. The red rectangle shows the range of trading value-induced similarity of all assets *i* and *j* in the portfolio (on average) against a single within-price similarity. Our proposed similarity ($S_{ij^{\prime }}$) is a secondary tool besides the conventional one (*S*_*ij*_). The results show that corresponding to a certain value for *S*_*ij*_, portfolios have a range of values for $S_{ij^{\prime }}$. This yields a better differentiation among the portfolios. The portfolios with lower $S_{ij^{\prime }}$ are less affected by the trading value-induced communities on the price layer.

In Fig 5, panel (a) and panel (c) respectively show the price layer (as the target layer) and the trading value layer (as the source layer) both without any community detection heatmap. Panel (b) and panel (d) respectively show the hierarchical clustering heatmaps on the price and the trading value layer.

The hierarchical clustering heatmap on panel (b) is obtained by using Eq 5 on the adjacency matrix of the price layer (target). Also, the hierarchical clustering heatmap on panel (d) is obtained by using Eq 5 on the adjacency matrix of the trading value layer (source). Using Eq 5 on the adjacency matrix of the trading value layer obtains the structural similarity matrix of the trading value layer. Each element in the structural similarity matrix shows how $i^{\prime }$ and $j^{\prime }$ are structurally similar. These values are used with hierarchical clustering on the trading value layer and form the clustering heatmap on the trading value layer. Assets with low structural similarity have a small distance on the hierarchical clustering heatmap. Conversely, assets with high structural similarity are close to each other in the hierarchical clustering heatmap.

Now using the adjacency matrices of both the trading value layer (source) and the price layer (layers), we use Eq 4 to obtain the node-wise interlayer self-similarity vector. Each element *i* of this vector denotes the interlayer self-similarity of asset *i*. It shows how the behavior of an asset *i* in making connections with other assets is similar across layers. Through interlayer self-similarity of node *i* ($S_{ii^{\prime }}$), the structural similarity of node *i* between the price and trading value layers (the two attributes of node *i*) is measured. Then, it is multiplied by the structural similarity ($S_{i^{\prime }j^{\prime }}$) between node $i^{\prime }$ and $j^{\prime }$ on the trading value layer (source). After calculation of Eq 6, a matrix of interlayer similarity ${𝒮}^{\left[\text{inter}\right]}=[S_{ij^{\prime }}]$ is obtained.

The elements of the interlayer structural similarity matrix, $S_{ij^{\prime }}$, contain the trading value-induced similarities on the price of assets. In panel (e) of Fig 2, we use hierarchical clustering on the price layer (target) by using the similarity matrix obtained from Eq 6, ${𝒮}^{\left[\text{inter}\right]}=S_{ij^{\prime }}$. At this point, hierarchical clustering arranges the assets with high (low) $S_{ij^{\prime }}$ closer (more distant).

Comparing the hierarchical clustering heatmaps of the price layer that one of them is obtained from *S*_*ij*_, panel (b), and the other one is obtained from $S_{ij^{\prime }}$, panel (e), shows that the hierarchical clustering heatmap based solely on the analysis of the price layer is different from the hierarchical clustering heatmap based on the trading value induced-effects into the price. Note that both panels (b) and (e) show the price return correlation clustering heatmap but with different structural similarities (Eqs 3 and 6). In panel (b), the clustering is derived from structural similarities on the price layer, and in panel (e), the clustering is derived from structural similarities that are induced by structural similarities of the trading value layer and the interlayer self-similarity (Eq 6). In these two community structures demonstrated in panel (b) and panel (e), the correlation values between price returns are the same, but the arrangement of assets next to each other is different because they result from two different structural similarity functions. Also, from a network perspective, one can see that the topology of the price layer network obtained from Eqs 3 and 6 are different. Such structures are not unique. Adding the trading value layer as a source of influence on the price layer leads to taking more information into account. This helps extract more information about the price layer. This method shows that the source of extracted hidden information about the price layer can be another layer, such as the trading value.

As shown, such information about the price network is not detectable by conventional community detection methods because the conventional methods seek to find communities based on the highlighted connections and nodes on the price layer. Our methodology shows that some dynamics on the price network stem from another layer such as the trading value. The trading value network spreads out interlayer systemic dynamics into the price network structure. Since this obtained information does not originate from the links on the price layer, the new structure is not observed from the eye of sole analysis of the price layer.

This research reveals that price and trading value layers in the multilayer networks may have some sort of interlayer interdependencies. While a system’s intrinsic nature is a multilayer network, one should be cautious about the influence of interdependent layers. Before applying our proposed methodology, one should not conclude that one can dismiss the multilayer effects in the financial network or networks with covert interlayer community structures. We find structures in the price and trading value multilayer network that one cannot observe in monolayer price return community detection because in conventional monolayer, the influence of $S_{ij^{\prime }}$ is not observable.

The topology of a link between two assets only within a layer is not always a good predictor of the presence of a link with the same topology between those two assets in other layers. Source-induced interlayer similarities close to zero contribute to fewer effects from the source layer to the target layer. If the price layer and the trading value are independent, they cannot impose interlayer structures. This methodology has potential applications in network systems. In the following, we apply the proposed methodology to investment portfolios.

**An empirical application: Portfolio diversification.** One of the most important applications of this model is the evaluation of network similarity in financial markets, such as investment portfolio diversification. These works focus solely on network of price returns to diversify portfolios, such as minimum spanning tree (MST) clustering [67], mutual information-based clustering [68], structural asset clusters using modularity [69], network-based tools and centrality [70], network-based risk parity allocation and minimum spanning trees [71]. However, price is not an isolated variable in financial markets, and other variables interact with price [55,66]. A diversified portfolio [72] is based on selecting assets that belong to less similar communities. Portfolio risk arises from the similarity between the assets within the portfolio. Thus, an important aspect of portfolio asset allocation is to scrutinize the price returns of the assets and their relationships. Assets should be allocated from assets with as few similar connections as possible. This results in allocating assets to the portfolio with more distant communities. It avoids high comovements among asset prices and leads to more diversified portfolios.

We suggest a financial application that utilizes the trading value layer as a secondary tool to gain a deeper understanding of the price layer structure. It enhances diversification strategies by capturing hidden structural influences on price returns. This approach builds on the idea of interlayer structural spillovers, where the trading value network imposes hidden community structures onto the price network. This secondary tool can be mounted on the main methods that only focus on price. This work suggests a tool to avoid over-concentration in assets that appear diversified based on price but exhibit hidden trading value-induced co-movements. Interestingly, our method provides a complementary perspective that conventional methods miss. Rather than replacing traditional diversification metrics, this approach adds an extra dimension to decision-making. A multi-method approach is often more effective in real-world portfolio construction.

Some literature also takes into account the influence of trading activities on portfolio construction. They show that overlapping portfolios (containing a portion of identical assets) impose risk on each other and can cause the financial system’s fragility. Because other investors’ cash flows (buying and selling decisions) can impose volatility on the price return. Since other portfolios may have that asset, such trading decisions transmit volatility from one portfolio to other portfolios in two ways: via the mutual assets [66], and also via assets that belong to the same community and exist in other portfolios. In other words, even if the portfolios do not have exactly the same assets, due to communities, a sudden and powerful trading activity and price change in an asset may influence other assets within that community. For diversification, apart from strong structures that are detectable by conventional community detection, one must be able to find the hidden community structures in the price layer that may originate from the trading value layer. Using this method, similarity can be calculated not only based on price return but also similarity due to the induced effects by other economic parameters, such as transaction value, which affects price return.

**Synthetic portfolios.** We construct 1000 equally-weighted synthetic portfolios, each of which contains 10 assets from the dataset. The low similarity of assets within a portfolio is an indication of a well-diversified portfolio. Portfolios with assets belonging to more distant clusters are less likely to spread and receive risk from each other and have less comovement.

To have a comparative analysis of the diversification power of the method, in Fig 5, we suggest the similarities within each portfolio in two dimensions. In Fig 5, the synthetic portfolios are plotted in the scatter plots. As mentioned, 10 assets are allocated from the dataset to construct the synthetic portfolios. We already have the structural similarity matrix $𝒮=S_{ij}$ within the price layer, and trading value-induced interlayer structural similarity on the price layer, ${𝒮}^{\left[\text{inter}\right]}=[S_{ij^{\prime }}]$. Thus, for each pair of assets *i* and *j* in a portfolio, we extract *S*_*ij*_ and $S_{ij^{\prime }}$. In the next step, we calculate the average of structural similarity values for each measurement in a portfolio.

The X-axes of Fig 5 are the average intralayer structural similarities of the price layer, ${\bar{S}}_{ij}$, for all assets *i* and *j* in a portfolio. It is calculated by Eq 11, as below:

Sij¯=1(Np2)∑i,jNpSij, (11)

A high $\bar{S_{ij}}$ denotes more likelihood that the price returns of assets in the portfolio get clustered together. This emphasizes higher co-behavior within prices in the portfolio.

The Y-axes in Fig 5 are the average interlayer structural similarities (trading value-induced effects on community structures on the price layer), ${\bar{S}}_{ij^{\prime }}$, for all assets *i* and *j* in a portfolio. It is calculated by Eq 12:

S¯ij′=1(Np2)∑i,j′NpSij′, (12)

A high $\bar{S_{ij^{\prime }}}$ denotes more likelihood that the price returns community structures of assets get affected and vulnerable by the trading activities of assets in the portfolio. The hidden community structures are not the dominant structures on the price layer (whereas if they are dominant, they are detectable by community detection solely based on the links on the price layer).

In the right panel of Fig 5, we magnify the scatter plot in an inset focusing on a specific value of ${\bar{S}}_{ij}$. Our objective is to demonstrate that for an identical value of ${\bar{S}}_{ij}$ (which only considers the price layer), there exist multiple ${\bar{S}}_{ij^{\prime }}$. This implies that among several portfolios with the same diversification on price ${\bar{S}}_{ij}$, their structural characteristics in terms of trading value-induced structures on price may differ. Therefore, this criterion can also be considered for diversification. Note that the color bars in Fig 5 denote the concept of “Equi-Diversification Curve”, which means that on this curve the diversification is equal. It is calculated by:

$${\bar{S}}_{ED}=\sqrt{\bar{S_{ij}^{2}}+{\bar{S}}_{ij^{\prime }}^{2}},$$ (13)

where each ${\bar{S}}_{ED}$ curve shows the locus of ${\bar{S}}_{ED}$ equal diversification. Portfolios with identical colors have the same aggregated diversification. Also, the angle bisector dashed line represents the locus of the equality of the two criteria.

The initial ideas of hierarchical clustering had size-effect issues which were not appropriate for large networks. Today, certain methods are suggested to alleviate the size-effect and scalability issues. Scalable Hierarchical Agglomerative Clustering [59] overcomes them by taking advantage of a nearest-neighbor graph-based merging approach, lowering the complexity and making billions of points clusterable. One limitation of our model is that it can be computationally expensive, especially for large datasets. In our work, we use hierarchical clustering for financial networks that are not too big. To enhance the scalability and performance of clustering, complex data clustering (CDC) [65] uses graph filtering and concentrates on single-layer or multi-view clustering. Multi-view attributed graph clustering (MAGC) [61] efficiently clusters multi-view attributed graphs using graph filtering and a consensus-based similarity learning approach, and primarily handles multiple attribute views within a single-layer clustering framework. Differently, our model uncovers the structural influence of one network layer on another, revealing hidden community structures. It captures interlayer dependencies, particularly in financial networks, social interactions, and covert organizational structures, where hidden influences play a crucial role in systemic behaviors. To address hierarchical clustering for large networks with sparse matrices, we note that SparseHC [73] offers an online agglomerative clustering approach that processes sorted distance data in chunks and uses an adjacency map for compact, constant-time access to active edges. This achieves sub-quadratic memory and near-quadratic time complexity even for very large sparse matrices. Inspired by this, our implementation can adopt similar sparse-aware data structures to ensure scalability.

## Conclusion

This research finds an internetwork systemic contagion via community structures imposed (or induced) by a source network into the target network. Other than node-to-node interactions, real-world networks influence each other via network-to-network interactions. Through network-to-network interactions, hidden structures spill over from the source network to the target network. These transmitted structures generate communities on the target network that conventional community detection methods on the target network cannot detect because the conventional community detection methods work based on the highlighted connections on the target network, but the origin of these structures stems from the source network, not the target network. Thus, these community structures are hidden and are not detectable by conventional community detection methods on the target layer. This phenomenon generates an internetwork hidden systemic contagion and forms covert groups on the target network–that are led from the source network.

Conventional diversification methods use price return similarity functions to define clusters and then allocate assets from more distant clusters. We provide a complementary approach for portfolio diversification to allocate assets from less structurally similar assets. Among synthetically generated portfolios from the real data, we obtained the average structural similarity of price (conventional tool) to compare it with the average trading-value-induced interlayer similarities on price. We find that for portfolios that have a specific value of within-price structural similarity, the portfolios may have a range of interlayer-induced similarities. This shows that these portfolios have different susceptibilities to trading activities. This methodology can be generalized to multilayer networks or multiple dependent networks.

Our model has significant implications for compliance purposes, anti-terrorism, anti-money laundering measures, detecting covert organizations, and systemic risk evaluation in financial-economic networks. For instance, some covert organizations, such as criminal organizations, money laundering, market manipulation, and terrorist groups, have two main layers: the source and the target. They hide their connections in the target layer (observable world) and then use the source layer (video game platforms) for managing and leading their cooperation in the target layer without leaving a trace for identification in the target layer. As another example, relationships with friends on different social networks may differ, but the quality of relationships on one platform can influence relationships on another. Many systems in the economy and finance, such as banks and firms, have heterogeneous connections. A layer can represent each type of connection. The structure of relationships in one layer may alter how those firms behave in another.

## Appendix

**A.1. Different cases in** Fig 3: For clarification purposes, we explain the calculation of case B as an example. For simplicity, we use binary links.

**Case B: Similarity calculations**

Assume we have the source and the target layers as below.

Considering the nodes, $i^{\prime }$, $j^{\prime }$, $\alpha^{\prime }$, $\beta^{\prime }$, $\gamma^{\prime }$, $\eta^{\prime }$, $\sigma^{\prime }$, $\tau^{\prime }$ on the source layer and their correspondence on the target layer, *i*, *j*, *α*, *β*, *γ*, *η*, *σ*, *τ*:

**Step 1:** Intralayer similarity *S*_*ij*_ of the target layer, and vectors for node *i* and node *j* are ${𝐯}_{i}=[0,0,1,1,1,0,0,0],{𝐯}_{j}=[0,0,0,0,0,1,1,1],n_{ij}={𝐯}_{i}\cdot {𝐯}_{j}=0,\|{𝐯}_{i}\|=\sqrt{3},\|{𝐯}_{j}\|=\sqrt{3}$.

Thus, $S_{ij}=\frac{{𝐯}_{i}\cdot {𝐯}_{j}}{\left\|{𝐯}_{i}\|\cdot \|{𝐯}_{j}\right\|}=\frac{0}{\sqrt{3}\cdot \sqrt{3}}=0$

**Step 2:** Intralayer similarity $S_{i^{\prime }j^{\prime }}$ of the source layer, vectors for node *i* and node *j*: ${𝐯}_{i^{\prime }}=[0,0,1,1,1,1,1,1],{𝐯}_{j^{\prime }}=[0,0,0,0,0,1,1,1],n_{i^{\prime }j^{\prime }}={𝐯}_{i^{\prime }}\cdot {𝐯}_{j^{\prime }}=3,\|{𝐯}_{i^{\prime }}\|=\sqrt{6},\|{𝐯}_{j^{\prime }}\|=\sqrt{3}$.

Thus, $S_{i^{\prime }j^{\prime }}=\frac{{𝐯}_{i^{\prime }}\cdot {𝐯}_{j^{\prime }}}{\left\|{𝐯}_{i^{\prime }}\|\cdot \|{𝐯}_{j^{\prime }}\right\|}=\frac{3}{\sqrt{6}\cdot \sqrt{3}}=\frac{1}{\sqrt{2}}\approx 0.707$

**Step 3:** $S_{ii^{\prime }}$ between layers, vectors for *i*: ${𝐯}_{i^{\prime }}=[0,0,1,1,1,1,1,1],\;{𝐯}_{i}=[0,0,1,1,1,0,0,0]$, ${𝐯}_{i^{\prime }}\cdot {𝐯}_{i}=3,\|{𝐯}_{i^{\prime }}\|=\sqrt{6},\|{𝐯}_{i}\|=\sqrt{3}$.

Thus, the interlayer self-similarity of node *i*, $S_{ii^{\prime }}=\frac{{𝐯}_{i^{\prime }}\cdot {𝐯}_{i}}{\left\|{𝐯}_{i^{\prime }}\|\cdot \|{𝐯}_{i}\right\|}=\frac{3}{\sqrt{6}\cdot \sqrt{3}}=\frac{3}{\sqrt{18}}=\frac{3}{3\sqrt{2}}=\frac{1}{\sqrt{2}}\approx 0.707$.

**Step 4:** For $S_{ij^{\prime }}$, we have: $S_{ij^{\prime }}=S_{ii^{\prime }}\cdot S_{i^{\prime }j^{\prime }}=S_{ij^{\prime }}=(0.707)(0.707)=0.5.$

Overall: $S_{ij}=0,\;S_{i^{\prime }j^{\prime }}=0.707,\;S_{ii^{\prime }}=0.707,\;S_{ij^{\prime }}=0.5$.

## References

[1] Hoffmann T, Peel L, Lambiotte R, Jones NS. Community detection in networks without observing edges. Sci Adv. 2020;6(4):eaav1478. doi: 10.1126/sciadv.aav1478 32042892 PMC6981088

[2] Aiyappa R, Flammini A, Ahn Y-Y. Emergence of simple and complex contagion dynamics from weighted belief networks. Sci Adv. 2024;10(15):eadh4439. doi: 10.1126/sciadv.adh4439 38608015 PMC11014438

[3] Lambiotte R, Ausloos M. Uncovering collective listening habits and music genres in bipartite networks. Phys Rev E Stat Nonlin Soft Matter Phys. 2005;72(6 Pt 2):066107. doi: 10.1103/PhysRevE.72.066107 16486010

[4] Gao J, Buldyrev SV, Stanley HE, Havlin S. Networks formed from interdependent networks. Nature Phys. 2011;8(1):40–8. doi: 10.1038/nphys2180

[5] Buldyrev SV, Parshani R, Paul G, Stanley HE, Havlin S. Catastrophic cascade of failures in interdependent networks. Nature. 2010;464(7291):1025–8. doi: 10.1038/nature08932 20393559

[6] Battiston S, Caldarelli G, D’Errico M. The financial system as a nexus of interconnected networks. Interconnected networks. Springer; 2016. p. 195–229.

[7] Kenett DY, Perc M, Boccaletti S. Networks of networks – an introduction. Chaos, Solitons & Fractals. 2015;80:1–6. doi: 10.1016/j.chaos.2015.03.016

[8] MacMahon M, Garlaschelli D. Community detection for correlation matrices. Phys Rev X. 2015;5(2):021006. doi: 10.1103/physrevx.5.021006

[9] Kivela M, Arenas A, Barthelemy M, Gleeson JP, Moreno Y, Porter MA. Multilayer networks. Journal of Complex Networks. 2014;2(3):203–71. doi: 10.1093/comnet/cnu016

[10] Boccaletti S, Bianconi G, Criado R, Del Genio CI, Gómez-Gardeñes J, Romance M, et al. The structure and dynamics of multilayer networks. Phys Rep. 2014;544(1):1–122. doi: 10.1016/j.physrep.2014.07.001 32834429 PMC7332224

[11] De Domenico M, Solé-Ribalta A, Cozzo E, Kivelä M, Moreno Y, Porter MA, et al. Mathematical formulation of multilayer networks. Phys Rev X. 2013;3(4). doi: 10.1103/physrevx.3.041022

[12] Egami N. Spillover effects in the presence of unobserved networks. Polit Anal. 2020;29(3):287–316. doi: 10.1017/pan.2020.28

[13] Financial Action Task Force (FATF). Virtual Currencies: Key Definitions and Potential AML/CFT Risks. 2014. https://www.fatf-gafi.org/media/fatf/documents/reports/Virtual-currency-key-definitions-and-potential-aml-cft-risks.pdf

[14] Klein M. Video games might matter for terrorist financing. 2024. [cited 2024 June 14]. https://www.lawfaremedia.org/article/video-games-might-matter-for-terrorist-financing#::text=Extremists

[15] Al-Rawi A. Video games, terrorism, and ISIS’s Jihad 3.0. Terrorism and Political Violence. 2016;30(4):740–60. doi: 10.1080/09546553.2016.1207633

[16] Richet J-L. Laundering money online: a review of cybercriminals methods. arXiv preprint 2013. https://doi.org/arXiv:1310.2368

[17] Mastroianni B. How terrorists could use video games to communicate undetected. 2015. [cited 2024 June 14]. https://www.cbsnews.com/news/how-terrorists-could-use-video-games-to-communicate-undetected/

[18] Gandica Y, Béreau S, Gnabo J-Y. A multilevel analysis of financial institutions’ systemic exposure from local and system-wide information. Sci Rep. 2020;10(1):17657. doi: 10.1038/s41598-020-74259-7 33077760 PMC7573582

[19] Nie C-X, Song F-T. Stable versus fragile community structures in the correlation dynamics of Chinese industry indices. Chaos, Solitons & Fractals. 2023;167:113044. doi: 10.1016/j.chaos.2022.113044

[20] Namaki A, Raei R, Ardalankia J, Hedayatifar L, Hosseiny A, Haven E, et al. Analysis of the global banking network by random matrix theory. Front Phys. 2021;8. doi: 10.3389/fphy.2020.586561

[21] Zahedian M, Bagherikalhor M, Trufanov A, Jafari GR. Financial crisis in the framework of non-zero temperature balance theory. PLoS One. 2022;17(12):e0279089. doi: 10.1371/journal.pone.0279089 36548258 PMC9779058

[22] Etesami J, Habibnia A, Kiyavash N. Modeling systemic risk: a time-varying nonparametric causal inference framework. arXiv preprint 2023. https://arxiv.org/abs/2312.16707

[23] Hu L, Gan Y, Wen H. Do we need to consider multiple inter-bank linkages for systemic risk in China’s banking industry? Analysis based on the multilayer network. Finance Research Letters. 2023;51:103433. doi: 10.1016/j.frl.2022.103433

[24] Cao J, Wen F, Stanley HE, Wang X. Multilayer financial networks and systemic importance: evidence from China. International Review of Financial Analysis. 2021;78:101882. doi: 10.1016/j.irfa.2021.101882

[25] Poledna S, Molina-Borboa JL, Martínez-Jaramillo S, van der Leij M, Thurner S. The multi-layer network nature of systemic risk and its implications for the costs of financial crises. Journal of Financial Stability. 2015;20:70–81. doi: 10.1016/j.jfs.2015.08.001

[26] Bazzi M, Porter MA, Williams S, McDonald M, Fenn DJ, Howison SD. Community detection in temporal multilayer networks, with an application to correlation networks. Multiscale Model Simul. 2016;14(1):1–41. doi: 10.1137/15m1009615

[27] Huang X, Chen D, Ren T, Wang D. A survey of community detection methods in multilayer networks. Data Min Knowl Disc. 2020;35(1):1–45. doi: 10.1007/s10618-020-00716-6

[28] Khawaja FR, Sheng J, Wang B, Memon Y. Uncovering hidden community structure in multi-layer networks. Applied Sciences. 2021;11(6):2857. doi: 10.3390/app11062857

[29] Martinet L-E, Kramer MA, Viles W, Perkins LN, Spencer E, Chu CJ, et al. Robust dynamic community detection with applications to human brain functional networks. Nat Commun. 2020;11(1):2785. doi: 10.1038/s41467-020-16285-7 32503997 PMC7275079

[30] Interdonato R, Tagarelli A, Ienco D, Sallaberry A, Poncelet P. Local community detection in multilayer networks. Data Min Knowl Disc. 2017;31(5):1444–79. doi: 10.1007/s10618-017-0525-y

[31] Karaaslanli A, Ortiz-Bouza M, Munia TTK, Aviyente S. Community detection in multi-frequency EEG networks. Sci Rep. 2023;13(1):8114. doi: 10.1038/s41598-023-35232-2 37208422 PMC10199028

[32] Dong G, Fan J, Shekhtman LM, Shai S, Du R, Tian L, et al. Resilience of networks with community structure behaves as if under an external field. Proc Natl Acad Sci U S A. 2018;115(27):6911–5. doi: 10.1073/pnas.1801588115 29925594 PMC6142202

[33] Ardalankia J, Askari J, Sheykhali S, Haven E, Reza Jafari G. Mapping coupled time-series onto a complex network. EPL. 2020;132(5):58002. doi: 10.1209/0295-5075/132/58002

[34] Anagnostou I, Squartini T, Kandhai D, Garlaschelli D. Uncovering the mesoscale structure of the credit default swap market to improve portfolio risk modelling. Quantitative Finance. 2021;21(9):1501–18. doi: 10.1080/14697688.2021.1890807

[35] Bargigli L, di Iasio G, Infante L, Lillo F, Pierobon F. The multiplex structure of interbank networks. Quantitative Finance. 2014;15(4):673–91. doi: 10.1080/14697688.2014.968356

[36] Solé-Ribalta A, Granell C, Gómez S, Arenas A. Information transfer in community structured multiplex networks. Frontiers in Physics. 2015;3:61.

[37] Newman MEJ. Detecting community structure in networks. The European Physical Journal B. 2004;38:321–30.

[38] Newman MEJ. Modularity and community structure in networks. Proc Natl Acad Sci U S A. 2006;103(23):8577–82. doi: 10.1073/pnas.0601602103 16723398 PMC1482622

[39] Gligor M, Ausloos M. Clusters in weighted macroeconomic networks: the EU case. Introducing the overlapping index of GDP/capita fluctuation correlations. Eur Phys J B. 2008;63(4):533–9. doi: 10.1140/epjb/e2008-00176-y

[40] Gligor M, Ausloos M. Convergence and Cluster Structures in EU Area according to Fluctuations in Macroeconomic Indices. JEI. 2008;23(2):297–330. doi: 10.11130/jei.2008.23.2.297

[41] Redelico FO, Proto AN, Ausloos M. Hierarchical structures in the gross domestic product per capita fluctuation in Latin American countries. Physica A: Statistical Mechanics and its Applications. 2009;388(17):3527–35. doi: 10.1016/j.physa.2009.05.033

[42] Newman MEJ. Communities, modules and large-scale structure in networks. Nature Phys. 2011;8(1):25–31. doi: 10.1038/nphys2162

[43] Radicchi F, Castellano C, Cecconi F, Loreto V, Parisi D. Defining and identifying communities in networks. Proc Natl Acad Sci U S A. 2004;101(9):2658–63. doi: 10.1073/pnas.0400054101 14981240 PMC365677

[44] Mantegna RN. Hierarchical structure in financial markets. Eur Phys J B. 1999;11(1):193–7. doi: 10.1007/s100510050929

[45] Liu J-G, Hou L, Pan X, Guo Q, Zhou T. Stability of similarity measurements for bipartite networks. Sci Rep. 2016;6:18653. doi: 10.1038/srep18653 26725688 PMC4698667

[46] Xie Y, Gong M, Wang S, Liu W, Yu B. Sim2vec: Node similarity preserving network embedding. Information Sciences. 2019;495:37–51. doi: 10.1016/j.ins.2019.05.001

[47] Talaga S, Nowak A. Structural measures of similarity and complementarity in complex networks. Sci Rep. 2022;12(1):16580. doi: 10.1038/s41598-022-20710-w 36195736 PMC9532398

[48] Nielsen F. Introduction to HPC with MPI for data science. Springer; 2016.

[49] Murtagh F, Contreras P. Algorithms for hierarchical clustering: an overview. WIREs Data Min & Knowl. 2011;2(1):86–97. doi: 10.1002/widm.53

[50] Tumminello M, Lillo F, Mantegna RN. Correlation, hierarchies, and networks in financial markets. Journal of Economic Behavior & Organization. 2010;75(1):40–58. doi: 10.1016/j.jebo.2010.01.004

[51] Lambiotte R, Rosvall M, Scholtes I. From networks to optimal higher-order models of complex systems. Nat Phys. 2019;15(4):313–20. doi: 10.1038/s41567-019-0459-y 30956684 PMC6445364

[52] Pryke A, Mostaghim S, Nazemi A. Heatmap visualization of population based multi objective algorithms. In: Evolutionary Multi-Criterion Optimization: 4th International Conference, EMO 2007, Matsushima, Japan, March 5-8, 2007. Proceedings. 2007. p. 361–75.

[53] Cerqueti R, Clemente GP, Grassi R. Stratified cohesiveness in complex business networks. Journal of Business Research. 2021;129:515–26. doi: 10.1016/j.jbusres.2020.04.005

[54] Lambiotte R, Ausloos M. On the genre-fication of music: a percolation approach. Eur Phys J B. 2006;50(1–2):183–8. doi: 10.1140/epjb/e2006-00115-0

[55] Ardalankia J, Osoolian M, Haven E, Jafari GR. Scaling features of price–volume cross correlation. Physica A: Statistical Mechanics and its Applications. 2020;549:124111.

[56] Civitarese J. Volatility and correlation-based systemic risk measures in the US market. Physica A: Statistical Mechanics and its Applications. 2016;459:55–67. doi: 10.1016/j.physa.2016.03.095

[57] Lambiotte R, Ausloos M. Collaborative tagging as a tripartite network. In: Computational Science–ICCS 2006 : 6th International Conference, Reading, UK, May 28-31, 2006. Proceedings, Part III, 2006. 1114–7.

[58] Lambiotte R, Ausloos M. Growing network with j-redirection. Europhys Lett. 2007;77(5):58002. doi: 10.1209/0295-5075/77/58002

[59] Monath N, Dubey KA, Guruganesh G, Zaheer M, Ahmed A, McCallum A, et al. Scalable hierarchical agglomerative clustering. In: Proceedings of the 27th ACM SIGKDD Conference on Knowledge Discovery & Data Mining. 2021. p. 1245–55. 10.1145/3447548.3467404

[60] Fortunato S. Community detection in graphs. Physics Reports. 2010;486(3–5):75–174. doi: 10.1016/j.physrep.2009.11.002

[61] Lin Z, Kang Z, Zhang L, Tian L. Multi-view attributed graph clustering. IEEE Trans Knowl Data Eng. 2021:1. doi: 10.1109/tkde.2021.310122736506788

[62] Lancichinetti A, Fortunato S, Kertész J. Detecting the overlapping and hierarchical community structure in complex networks. New J Phys. 2009;11(3):033015. doi: 10.1088/1367-2630/11/3/033015

[63] Fortunato S, Barthélemy M. Resolution limit in community detection. Proc Natl Acad Sci U S A. 2007;104(1):36–41. doi: 10.1073/pnas.0605965104 17190818 PMC1765466

[64] Girvan M, Newman MEJ. Community structure in social and biological networks. Proc Natl Acad Sci U S A. 2002;99(12):7821–6. doi: 10.1073/pnas.122653799 12060727 PMC122977

[65] Kang Z, Xie X, Li B, Pan E. CDC: a simple framework for complex data clustering. IEEE Transactions on Neural Networks and Learning Systems. 2024. 10.1109/TNNLS.2024.3473618 39401105

[66] Delpini D, Battiston S, Caldarelli G, Riccaboni M. Portfolio diversification, differentiation and the robustness of holdings networks. Appl Netw Sci. 2020;5(1). doi: 10.1007/s41109-020-00278-y

[67] Ren F, Lu Y-N, Li S-P, Jiang X-F, Zhong L-X, Qiu T. Dynamic portfolio strategy using clustering approach. PLoS One. 2017;12(1):e0169299. doi: 10.1371/journal.pone.0169299 28129333 PMC5271336

[68] Sharma C, Habib A. Mutual information based stock networks and portfolio selection for intraday traders using high frequency data: An Indian market case study. PLoS One. 2019;14(8):e0221910. doi: 10.1371/journal.pone.0221910 31465507 PMC6715228

[69] Ferretti S. On the modeling and simulation of portfolio allocation schemes: an approach based on network community detection. Comput Econ. 2022;62(3):969–1005. doi: 10.1007/s10614-022-10288-w

[70] Clemente GP, Grassi R, Hitaj A. Smart network based portfolios. Annals of Operations Research. 2022;316(2):1519–41. 35431386 10.1007/s10479-022-04675-7 PMC8995926

[71] Ciciretti V, Pallotta A. Network risk parity: graph theory-based portfolio construction. J Asset Manag. 2024;25(2):136–46. doi: 10.1057/s41260-023-00347-8

[72] Koumou GB. Diversification and portfolio theory: a review. Financ Mark Portf Manag. 2020;34(3):267–312. doi: 10.1007/s11408-020-00352-6

[73] Nguyen T-D, Schmidt B, Kwoh C-K. SparseHC: a memory-efficient online hierarchical clustering algorithm. Procedia Computer Science. 2014;29:8–19. doi: 10.1016/j.procs.2014.05.001

